# Electromagnetic Navigation Bronchoscopy in Hybrid Theater

**DOI:** 10.3389/fsurg.2019.00010

**Published:** 2019-03-19

**Authors:** Ze-Rui Zhao, Rainbow W. H. Lau, Calvin S. H. Ng

**Affiliations:** ^1^State Key Laboratory of Oncology in Southern China, Department of Thoracic Surgery and Collaborative Innovation Centre for Cancer Medicine, Sun Yat-Sen University Cancer Centre, Guangzhou, China; ^2^Division of Cardiothoracic Surgery, Department of Surgery, Prince of Wales Hospital, The Chinese University of Hong Kong, Shatin, China

**Keywords:** electromagnetic navigation bronchoscopy, hybrid operation room, localization, radiofrequency ablation, photodynamic therapy

## Introduction

Electromagnetic navigation bronchoscopy (ENB) was designed to extend bronchoscopy to the peripheral airways beyond where can be approached via traditional bronchoscopy by using software-based reconstructed virtual airway and hardware-based real-time navigation. When compared with traditional bronchoscopy, ENB significantly increases the overall diagnostic yield in the biopsy of peripheral pulmonary lesions (1).

To deploy biopsy tools or conducting other transbronchial interventions, the extended working channel (EWC) of ENB should be fixed in a position toward the target lesion. Various adjuvant techniques can be used to verify the direction of EWC during ENB. Fluoroscopy is generally used in reported series to examine the position of the EWC, whereas some lesions, especially those with ground-glass opacity can be hard to visualize during X-ray. Linear endobronchial ultrasound is usually adopted when simultaneous lymph node assessment is required or dealing with more challenging cases. In a recently published prospective, multicenter study evaluating ENB for peripheral pulmonary lesions (the NAVIGATE trial), fluoroscopy, endobronchial ultrasound, and cone-beam CT was used in 91, 57.4, and 4.9%, respectively (2). Notably, the application of the hybrid operating room (OR) in the minimally invasive thoracic procedure is rapidly gaining acceptance in recent years (3). The incorporated DynaCT within the hybrid theater provides unparalleled real-time images in high resolution which could improve location confirmation and visualization during ENB navigation. In the current mini-review, we discussed the ENB-guided pulmonary nodule biopsy and localization technique, as well as transbronchial local treatment in hybrid OR.

## Targeting Lesion via ENB in Hybrid OR: General Aspects and Technical Considerations

After general anesthesia and single-lumen endotracheal tube insertion, the patient is positioned in supine with the electromagnetic board underneath. The bronchoscope is then advanced to the segmental bronchus. Once there, the electromagnetic locatable guide is manipulated toward the target lesion under navigational pathway with virtual 3D airway based on preoperative CT scan on the ENB console screen. The EWC will be locked and held still, while the biopsy tool is deployed with the DynaCT scan revealing the precise direction of the biopsy needle. If there is a mal-position, the needle should be adjusted according to the real-time CT images. A second-round intraoperative CT scan can be performed if needed, so as to guarantee the biopsy needle is within the lesion by viewing the sagittal and coronal images (4). If a subsequent lung resection is required, the single-lumen endotracheal tube will be replaced with a double-lumen one and the patient will be placed in the lateral decubitus position. Once the EWC deviates from the target lesion, repeated biopsy/brushing can hardly increase the diagnostic yield of ENB. Hence, the advantage of performing ENB procedure in a hybrid OR is to overcome such “mis-hit” phenomena inherent from the navigation error, CT-body divergence, and tool deflection, hence increases the accuracy and effectiveness of ENB procedure in diagnosing small lesions.

Several technical aspects should be considered for using the ENB system (Medtronic superDimension Navigation System, Covidien, Minneapolis, MN, USA) in the hybrid OR environment:

1. The compatibility of the hybrid OR and the electromagnetic interference should be calibrated in advance;
2. It is recommended to disconnect the electromagnetic board prior to the DynaCT scan to preserve the image quality;
3. In order to avoid any dislodgement of the EWC and tool during CT scanning, the bronchoscope should be fixed by a non-metallic table-mounted device.

It should be noted that other navigational system such as the SPiN Thoracic Navigation System (Veran Medical Technologies, St. Louis, MO, USA) does not require the OR to be pre-mapped for the system and the specific procedure could be changed accordingly.

## ENB-guided Dye/Contrast Labeling for Assisting Pulmonary Resection

Compared with the conventional percutaneous approach, localizing tiny pulmonary nodules by dye/contrast injection through ENB route can significantly reduce the chance of dye diffusion and pneumothorax because the pleura is not typically breached. In a typical methylene blue-assisted technique performed in a conventional OR, a small dose of methylene blue (0.5–1 ml) is injected directly into the lesion if the nodule is located nearby the pleura under ENB guidance. For those located more than 5 to 10 mm from the pleura, the dye can be released at the midway between the lesion and the nearest pleural surface, or the double dye release approach can be adopted (at the lesion and at the pleural surface).

Several groups have reported results of ENB-guided dye-marking with methylene blue or indigo carmine followed by pulmonary resection with neglectable ENB-related complication rate (5–9). The success rate ranged from 79 to 100% in these reports after ENB dye localization. Interestingly, the diameter and the distance from the lesion to the pleural surface did not seem to affect the success rate of localization (9).

In a study by Krimsky et al. 21 patients received thoracoscopic or robotic-assisted thoracic surgery with ENB labeling using either methylene blue (*n* = 11) or indigo carmine (*n* = 10) regimen (6). Interestingly, 19% of the nodules (*n* = 4) experienced unsuccessful marking. One patient had diffuse dye marker staining of the parietal pleura and in other three patients, no dye marker was visible within the hemithorax. Similar unsuccessful episodes were seen in Munoz-Largacha et al. (10) and Marino et al. (9), mainly due to extravasation of dye into the pleural cavity or lung parenchyma.

**Figure 1:**
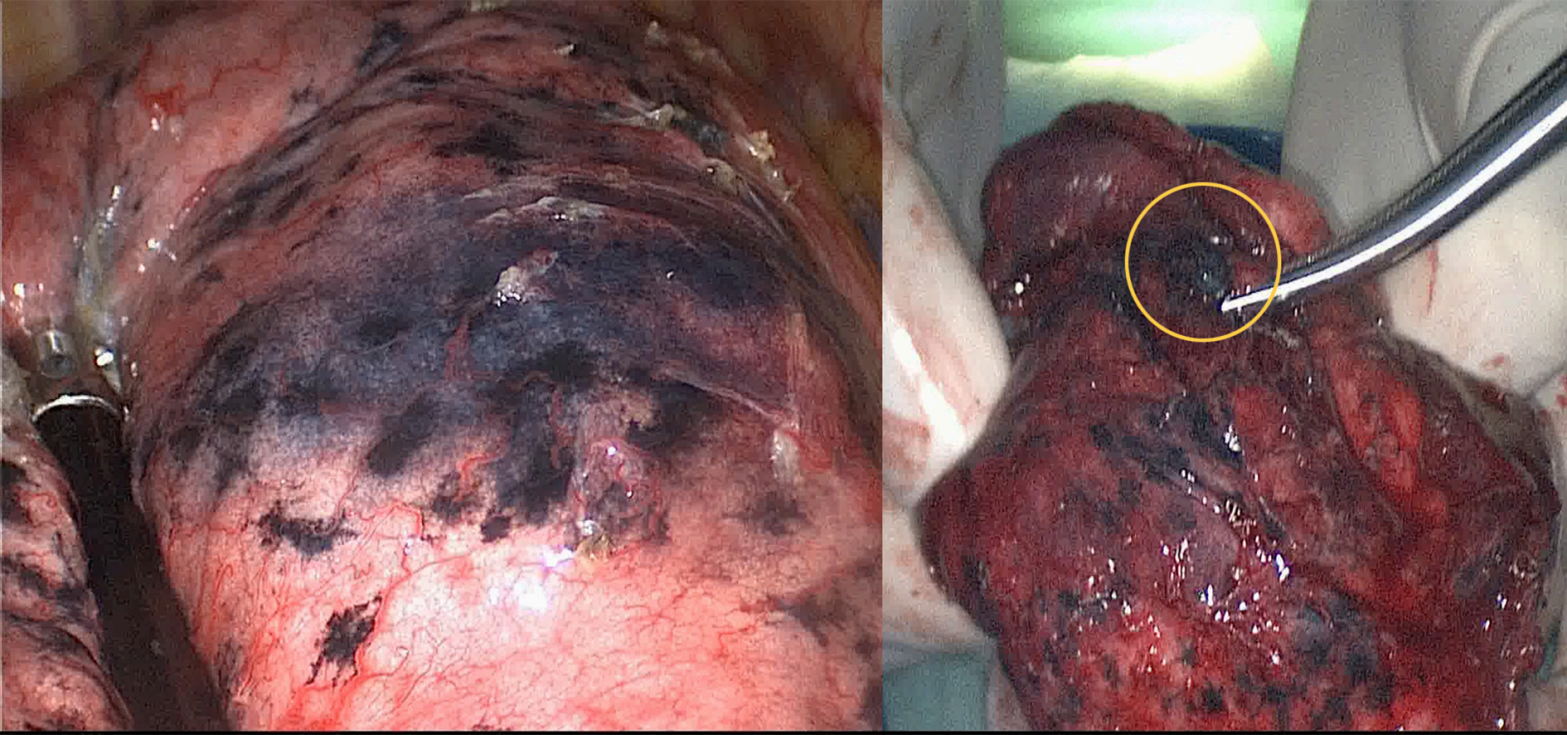
Electromagnetic navigation bronchoscopy-guided methylene blue plus fibrin sealant created a gelatinous appearance with visible stain (left side); the stain kept limited in the resected specimen after 2 h of operation (highlighted in a circle, right side).

To minimize the chance of dye dispersion, Luo et al. (11) invented a new method to maintain the location of ink by mixing methylene blue with fibrin sealant, which would create a gelatinous appearance with visible stain as well as tactile sense. In a recent case of our practice, the ENB-guided methylene blue injection help to guide a right S1a wedge resection of a 4 mm lesion combined with an anatomic S2 segmentectomy of a 12 mm ground-glass opacity. Moreover, the stained area remained clearly visible and well-demarcated after 120 min from the initial injection (Figure 1).

In patients with a heavy smoking history, pigmented deposits in the pleural surface could influence the intraoperative visualization of methylene blue. For this reason, indocyanine green (ICG) injection via ENB route has the advantage of superior dye visualization properties when viewed under near-infrared light, hence the marked target can be found regardless of the background color of the lung. The fluorescence emitted by ICG injected at a depth up to 20 mm from the pleural surface can be detected according to a previous study (12). In a recent study by Anayama et al. (13), 21 of the 22 patients who underwent ICG injection by ENB was detected by near-infrared thoracoscopy. Moreover, in 6 patients who underwent marking at two sites, 5 of them were detected successfully.

Dye injection may be less ideal for cases of severe pulmonary emphysema because the liquid markers do not form a confined spot. Therefore, it is reasonable that different techniques can be combined. For example, if the virtual bronchoscopy does not show appropriate airway leading to the lesion for dye-marking under ENB planning. Other adjuvant localizing methods can be considered. Our group had shown that percutaneous hookwire insertion and ENB dye-marking for different metastatic lesions is feasible and can be implemented in the hybrid OR as a one-stop approach (14). Notably, such a streamlined workflow within the hybrid OR would minimize the chance of dye diffusion and wire dislodgement.

**Figure 2:**
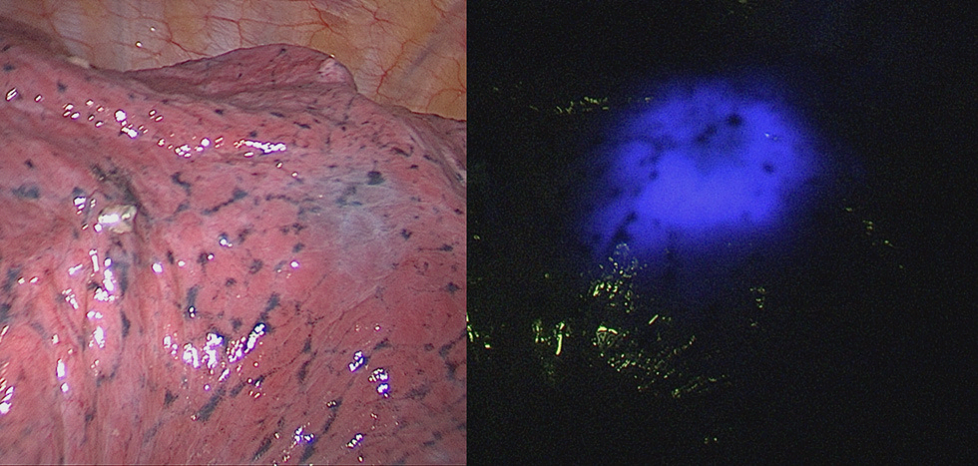
Thoracoscopic view of ENB methylene blue dye marked lesion that is difficult to identify **(left side)**, clearly visible fluorescence from ICG dye with near-infrared light for the same marked lesion **(right side)**.

To further expand the success rate of dye-marking, we recently develop a triple contrast dye-marking technique via ENB approach in the hybrid OR (15). The regimen includes the mixture of standard iohexol contrast, methylene blue, and ICG. As a result, the iohexol allow real-time visualization of dye-injection to the lesion via Dyna-CT scan. During the subsequent thoracoscopic procedure, methylene blue tattoo would guide the location of the lesion. In case of substantial pleural adhesiolysis, anthracotic lungs or other scenarios when methylene blue cannot be identified, the near-infrared light can localize the ICG as a backup strategy (Figure 2).

In centers without hybrid OR facilities, surgeons would generally send the patient to receive percutaneous hookwire insertion or dye injection in the morning and then operate on the case later in the day. In contrast, ENB labeling technique in the hybrid OR provides flexible OR scheduling for thoracic surgeons. Interestingly, if a definite diagnosis of lung cancer is made by rapid on-site pathologic evaluation via ENB, the surgeon can proceed directly to anatomic resection, rather than localization, wedge resection, and then anatomic resection in a routine manner. This would be important when treating multiple pulmonary lesions in suspicious of synchronous lung cancer.

## ENB-based Alternative Therapy in Hybrid OR

Alternative local therapy is an essential supplement treatment modality for early-stage lung cancers or metastatic lesions that are deemed unsuitable for operation due to severe comorbidities or limited pulmonary function. Emerging evidence shows that catheter-based therapies, such as radiofrequency ablation (RFA), microwave ablation, cryoablation, and photodynamic therapy (PDT) are feasible and safe, with manageable morbidities and toxicities (16). The advancement of instrument allows interventional therapy such as RFA, which is generally approached under percutaneous access associated with a higher chance of complications, to be delivered into periphery lung lesions via endobronchial route (17). In the study by Koizumi et al. (18), tumor control via CT-guided bronchoscopy cooled RFA was comparable to the percutaneous route in treating 23 peripheral stage I lung cancers. Only three patients developed an acute ablation-related reaction (fever, chest pain) and required hospitalization but improved with conservative treatment. Control rate was 82% at 6 months (partial response 48%, stable disease 34%). Twelve lesions had to be retreated during follow-up.

In a recent study, Xie et al. (19) reported the use of ENB to guide RFA in three patients of stage IA NSCLC or lung metastasis with a bronchus sign on CT scans. Interestingly, two patients achieved 1-year progression-free survival afterwards. Dedicated flexible electrode developed for bronchoscopic RFA can be applied for lesions up to 30 mm. It is plausible that ENB, as an advanced bronchoscopy, could enable more peripherally located lesions to be accurately reached for ablation treatment within the hybrid suite setting (4). In addition, the development of endobronchial microwave ablation could further improve ablation efficacy of these lung lesions.

PDT is another choice of alternative treatment for tiny lung malignancies. After the injection of a photosensitizer, laser light with a defined spectrum would activate local necrotic effect, which can achieve a 10 mm necrotic area on a swine model (20). The technique was originally used for a superficial and proximal tumor in lung cancer. With the aid of ENB, it is possible that light irradiation can be given to peripheral lesions in high accuracy. In the study by Chen et al. three pulmonary nodules with a mean size of 21.3 mm successfully underwent PDT via ENB (21). The position of the probe to deliver PDT irradiation was confirmed by Dyna-CT in the hybrid OR. Significantly tumor shrinkage was confirmed in all the patients during the follow-up CT scan (including two partial and one complete response). Regarding the procedure-related complication, only one patient experienced skin hypersensitivity 1 month after the PDT treatment.

## Perspective and Conclusion

As the specialty of interventional pulmonary is expanding, thoracic surgeons today need to continue to work in the field of advanced bronchoscopy to make certain the new technologies and interventions can be done by themselves. Compared with a conventional percutaneous approach, ENB has the advantages of reduced risk of pneumothorax and precise localization of lung tumor. Through a working channel, various marking dyes can be adopted to assist minimally invasive surgery. Additionally, ENB route allows different ablation techniques to be used in peripheral lung malignancies. The utilization of hybrid OR permits confirmation of the working probe location by real-time image, hence, making the navigation even more accurate. It is also possible that the endobronchial ablation process and the ablation zone can be monitored immediately in a hybrid suite. It is plausible that surgeons would be able to choose different localization techniques and resection/ablation simultaneously without the delay which happens in traditional clinical pathways where procedures are performed in several locations (22).

However, potential challenges remain regarding the hybrid OR combined with ENB. Apart from its high implementation and maintenance cost, the main drawback is the increased radiation exposure to the patient and medical staff. Besides, the diagnostic yield of ENB for lower lobe lesions could drop significantly due to the navigation error arise from diaphragmatic breathing (23). Theoretically, general anesthesia as well as intraoperative CT scan in the hybrid OR would improve the chance of positive localization and biopsy for lower lobe lesions under ENB. Nevertheless, further evidence is warranted.

## Author Contributions

CN and Z-RZ contributed to conception and design. CN contributed to administrative support. Z-RZ and RL contributed to provision of study materials or patients. ZR-Z and CN contributed to collection and assembly of data. Z-RZ contributed to data analysis and interpretation. All authors contributed to manuscript writing and final approval of manuscript.

### Conflict of Interest Statement

CN has an electromagnetic navigation bronchoscopy system SuperDimension Version 7 on loan from Medtronic; and is a consultant for Siemens Healthineers. The remaining authors declare that the research was conducted in the absence of any commercial or financial relationships that could be construed as a potential conflict of interest.
